# Diabetes as a predictive factor for severe form and high mortality risk of COVID-19: Retrospective cohort study of 188 cases

**DOI:** 10.1016/j.amsu.2021.103095

**Published:** 2021-11-23

**Authors:** Sara Berrajaa, Samia Berrichi, Zakaria Bouayed, Sanae El Mezzeoui, Fatima Zahra Aftiss, Houssam Bkiyar, Naima Abda, Brahim Housni

**Affiliations:** a Anesthesia and Resuscitation Department, MOHAMMED VI University Hospital Center, Oujda, Morocco; b Laboratory of Epidemiology, Clinical Research and Public Health LERCSP, Faculty of Medicine and Pharmacy, Oujda, Morocco; c Simulation Center, Faculty of Medicine and Pharmacy, Oujda, Morocco

**Keywords:** COVID-19, Diabetes, Mortality, Predictive factors, Sevirity

## Abstract

**Introduction:**

Since the appearance of the first case of the SARS CoV 2 infection, several studies have been conducted to identify the predictive factors of mortality in patients with COVID-19. According to previous reports, diabetes seems to be associated with severe clinical forms of the new coronavirus (SARS CoV 2).

Our study aimed to identify the epidemiological, clinical, radiological and prognostic profile of diabetic patients with COVID-19.

**Methods:**

This retrospective study included diabetic patients diagnosed with COVID-19 and admitted to the Resuscitation Department of our university hospital center From Mars 1st 2020, to December 31st, 2020.

**Results and discussion:**

In this study, we collected the data of 600 patients admitted to the Anesthesia and Resuscitation Department of the Mohammed VI University Hospital of Oujda, a group of 188 (31.3%) had diabetes.

The median age of our patients was 67 [25–75]. Were noted in the majority, of patients 69.6% with diabetes have developed a severe or critical injuries in the Chest CT Scan. Furthermore, we found that the mortality rate in this category of patients was higher 65/188 (34.60%) compared to non-diabetic patients, 130/412 (31.60%) (34.60%vs 31.60%; p: 0.464).

**Conclusion:**

Based on the results of this retrospective study, we concluded that diabetes is predictive factor for the need of an intensive care as well as a high risk of mortality related to COVID-19.

Practically speaking, diabetic patients should be monitored more closely and need an aggressive preventive management protocols in order to prevent severe forms of the disease and a drastic evolution.

More research is direly needed to identify patients of a higher risk of developing severe forms of COVID-19.

## Highlights

• Several studies addressed a variety of factors linked to higher risk of severe forms of COVID-19 and a higher mortality rate.
• Diabetic patients are exposed to a variety of complications related to COVID-19.
• Predictive factors of severity and mortality such as diabetes help identify high-risk patients in need of an intensive care.

## 1 Introduction

As of December 2019, the COVID-19 caused by the severe acute respiratory syndrome coronavirus 2 (SARS-CoV-2) was initially identified in Wuhan, China and has rapidly become a global pandemic [1,2].

Several studies have shown that diabetes is associated with severe forms and complications of COVID-19: such as acute respiratory distress syndrome (ARDS), admission to the intensive care unit (ICU), use of mechanical ventilation, as well as a high mortality rate.

This chronic condition increases the risk of disabling and life-threatening complications from micro- and macro-vascular diseases [3]. Which is important to understand the special aspects of COVID-19 infection in people with this underlying comorbidity [4].

This study was conducted for the purpose of identifying the epidemiological, clinical, radiological and prognostic profile of diabetic patients with COVID-19 managed at the Anesthesia and Resuscitation Department of Mohammed VI University Hospital of Oujda -Morocco.

## 2 Objectives

The objective of this 10-months long retrospective study was to determine whether diabetes is a predictive factor for the occurrence of severe forms and high rate mortality related to COVID-19 in our department.

## 3 Materials and methods

### 3.1 Type of study

We conducted a retrospective cohort study, mono-centric, of diabetic patients with SARS CoV 2 pneumonia hospitalized in the Anesthesia and Resuscitation Department of the Mohammed VI University Hospital of Oujda during a ten months period, from March the 1st to December the 31st, 2020.

### 3.2 Inclusion criteria

Among the 600 patients gathered, a 188 had diabetes in this study were hospitalized, and diagnosed according to the World Health Organization (WHO) provisional guidelines.

Cases infected with SARS-CoV-2 were confirmed by reverse transcription-polymerase chain reaction (RT-PCR) testing of throat and nose samples or Chest CT Scan typically for COVID-19 during the study period spread over 10 months, between March 2020 and December 2020 were included in the study.

### 3.3 Data collection

Patient's baseline characteristics were noted in the time of admission (day zero) such as medical history, degree of pulmonary involvement, type of oxygen supplementation as well as the outcome.

The term “death due to COVID-19” according to WHO: “is a death resulting from a clinically compatible disease, in a probable or confirmed case of COVID-19, in the absence of any other obvious cause of death unrelated to the coronavirus disease” this definition was used in this work.

### 3.4 Statistical analysis

Statistical analysis was conducted using the SPSS version 25.0. to determine the most powerful factors, such as demographic data, including age and gender, and underlying diseases affecting the mortality outcome among COVID-19 patients with diabetes, and with other comorbidities [5].

### 3.5 Ethical approval and consent

This study does not require a formal ethical committee approval. Access to the data was authorized and approved by the head of Department.

Given the retrospective design of this study, the requirement of patient consent is lifted. Data anonymity was respected in accordance with national and international guidelines.

Our study was registered in Research Registry under the number: 7171.

This work has been reported in line with the STROCSS criteria [11].

## 4 Results

Six hundred (600) cases were admitted to our department, among them a 188 (31.3%) diabetic patients were included after fulfilling inclusion criteria.

### 4.1 Demographic and comorbidity data

The median of age was 67 [25–75] years, of which a 130 (69.1%) were males, and 58 (30, 9%) were females.

Pre-existing comorbidities were as follow: hypertension, 101 (53.7%), cardio vascular diseases 44 (23.4%), obesity 40 (21.3%), renal failure 19 (10.1%), Gout 12 (6.4%), hypothyroidism 9 (4.8%), hemopathy 3 (1%)

Patient's clinical characteristics at the admission are represented in1 Table 1

**Table 1:** Basic characteristics.

Variable	N %	
Age (mediane)	**67 (25**–**75)**	
Gender		
Male	**130**	**69.1%**
Female	**68**	**30.9%**
Comorbidity		
Hypertension	**101**	**53,7%**
Cardio vascular diseases	**44**	**23,4%**
Obesity	**40**	**21,3%**
Renal failure	**19**	**10,1%**
Gout	**12**	**6,4%**
Hypothyroïdisme	**9**	**4.8%**
Hemopathy	**3**	**1.6%**

**Table 2:** Types of oxygen supplementation at the admission.

Types of oxygen supplementation	*N*	*%*
oxygen cannula	108	**57,4**
HCM	162	**86.2**
OPTIFLOW	100	**53.2**
CPAP	19	**10.1**
NIV	49	**26.1**
MECHANICAL VENTILATION	59	**31.4**

### 4.2 Ventilation support for the patients

All our patients required oxygen supplementation with: oxygen cannula 108 (57.4%), high concentration oxygen mask (HCM) 162 (86.2%), high flow nasal oxygen therapy (OPTIFLOW) 100 (53.2%), CPAP ventilation 19 (10.1%), non-invasive ventilation(NIV) 49 (26.1%), and mechanical ventilation 59 (31.4%).

### 4.3 Degree of pulmonary involvement in chest CT

In terms of imaging, Chest CT was performed in 98,9% of cases, allowing the staging of our patients according to the degree of pulmonary involvement into 4 groups (represented in Table 3):

• 18 (9.6%) patients with minimal involvement of COVID-19 : 10–25%
• 37 (19.7%) patients with moderate involvement of COVID-19 : 25–50%
• 64 (34%) patients with severe involvement of COVID-19 : 50–75%
• 67 (35.6%) patients with critical involvement of COVID-19 : 75–100%

**Table 3:** Degree of pulmonary lesions.

Degree of pulmonary lesions	***N***	***%***
10–25%	18	**9.6**
25–50%	37	**19.7**
50–75%	64	**34**
75–100%	67	**35.6**

### 4.4 Complications and outcome

Among the 188 diabetic patients included in our study, 65 out of 188 (34.6%) died.

Furthermore, in our study we found that the evolution of diabetic patients hospitalized in the Anesthesia and Resuscitation Department of the Mohammed VI University Hospital of Oujda was characterized by the occurrence of multiple complications (represented in Table 4), represented as follows:

• Acute Kidney Failure: 85 (45.2%)
• Ischemia: 43 (22.9%)
• Septic shock: 39 (20.7%)
• Thrombocytopenia: 32 (17%)
• Pulmonary Embolism: 16 (8.5)
• Disseminated intravascular coagulation (DIC): 15 (8%)
• Cerebrovascular accident: ischemic stroke [3 (1.6%)], hemorrhagic cerebrovascular accident [1 (0.5%)]
• Pleural and pericardial effusions: 27 (13.8%)
• Myocarditis: 9 (4.8%)

**Table 4:** The most common complications.

Complication	***N***	***%***
Acute kidney failure	85	**45.2**
Ischemia	43	**22.9**
Septic shock	39	**20.7**
Thrombocytopenia	32	**17**
Pulmonary Embolism	16	**8.5**
Disseminated intravascular coagulation	15	**8**
Cerebrovascular accident	4	**2.1**
Pleural and pericardial effusions	27	**13.8**
Myocarditis	9	**4.8**

## 5 Discussion

COVID-19 is a global health crisis, challenging the preparedness of health systems’ all over the world as well as the ability to cope with and sustain a pandemic response [3]. It has been clinically observed that diabetes was associated with an increased risk of developing severe forms of COVID-19 such as ARDS (acute respiratory distress syndrome) and increased mortality risk.

Many studies conducted during the recent crisis, reported that diabetes plays a critical role in the outcome of SARS-CoV-2 pneumonia. According to a Chinese data on more than 70,000 cases, the overall mortality linked to COVID-19 was 2.3% versus 7.3% in diabetic patients [6]. In the study of Guo et al. [7], diabetic patients died much more often than non-diabetic patients (10.8% versus 3.6%).

In some other studies, it has been pointed that the COVID-19 mortality risk in pre‐ existing diabetes is 5‐ fold higher compared to individuals with normal glucose levels, while this is 10-fold higher in newly diagnosed patients [8].

In Italy, 35% of the deceased patients were diabetic, compared to 20% of the general population in this age group: therefore; diabetics are overrepresented among the deceased patients; 70% are men with average age of 80 years old [9].

According to our study, among the 600 cases admitted to our department, a 188 (31.3%) patients were diabetics with a higher mortality rate of 65/188 (34.60%), compared to non-diabetic patients with 130/412 deaths (31.60%). (34.60%vs 31.60%; p: 0.464).

In our group, 67 (35.6%) diabetic patients had critical injuries between 75 and 100% in the Chest CT, performed during their hospitalization.

We found that diabetes was often associated with other comorbidities, such as hypertension, 101 (53.7%), cardio vascular diseases 44 (23.4%), obesity 40 (21.3%), renal failure 19 (10.1%).

In addition, in this series of cases, we concluded that diabetic patients developed several complications characterized mainly by the occurrence of acute renal failure with a percentage of 45.2%, Ischemic accidents with a rate of 22.9%, septic shock found in 20.7% of our patients, and Thrombocytopenia in 17% of cases.

Furthermore, in a study conducted by Li et al., diabetes constitutes the highest percentage in terms of admission to the ICU and require more frequently mechanical ventilation [10].

These results are similar to those of our study, All our patients required oxygen supplementation where 49 (26.1%) benefited from oxygenation by non-invasive ventilation (NIV) and 59 (31.4%) of diabetes patients were intubated.

## 6 Conclusion

While diabetes does not appear to increase the incidence of COVID-19 infection, once infected, diabetic patients have an increased risk of developing severe forms of the disease thus requiring special attention.

From our study, we can conclude that diabetic patients developed more severe pulmonary lesions, requiring the use of non-invasive ventilation or intubation with a higher mortality rate than non-diabetic patients.

Practically speaking, diabetic patients should be monitored more closely and need an aggressive preventive management protocols in order to prevent severe forms of the disease and a drastic evolution.

## Provenance and peer review

Not commissioned, externally peer-reviewed.

## Funding

This research did not receive any form of funding

## Ethical approval

Research studies involving patients require ethical approval. Please state whether approval has been given, name the relevant ethics committee and the state the reference number for their judgement.

This study does not require a formal ethical committee approval.

## Consent

Given the retrospective design of this study, the requirement of patient consent is lifted. Data anonymity was respected in accordance with national and international guidelines.

## Author contribution

Sara BERRAJAA: Study concept, Data collection; data analysis; writing review & editing. Samia BERRICHI: Contributor. Zakaria BOUAYED: Contributor. Sanae EL MEZZEOUI: Contributor. Fatima Zahra AFTISS: Contributor. Houssam BKIYAR : supervision and data validation. Naima ABDA: Data collection; data analysis, editing, supervision and data validation. Brahim HOUSNI: supervision and data validation.

## Guarantor

The Guarantor is the one or more people who accept full responsibility for the work and/or the conduct of the study, had access to the data, and controlled the decision to publish. Sara BERRAJAA.

## Declaration of competing interest

We have no conflicts of interest.
